# Vitamin C supports conversion of human γδ T cells into FOXP3-expressing regulatory cells by epigenetic regulation

**DOI:** 10.1038/s41598-020-63572-w

**Published:** 2020-04-16

**Authors:** Léonce Kouakanou, Christian Peters, Qiwei Sun, Stefan Floess, Jaydeep Bhat, Jochen Huehn, Dieter Kabelitz

**Affiliations:** 1 Institute of Immunology, Christian-Albrechts-University Kiel, D-24105 Kiel, Germany; 2 BGI Genomics Institute, Shenzhen, China; 3 Experimental Immunology, Helmholtz Centre for Infection Research, D-38124 Braunschweig, Germany; 4 Metabolic Programming, School of Life Sciences, Technical University Munich (TUM), 85354 Freising, Germany

**Keywords:** Methylation analysis, Gammadelta T cells

## Abstract

Human γδ T cells are potent cytotoxic effector cells, produce a variety of cytokines, and can acquire regulatory activity. Induction of FOXP3, the key transcription factor of regulatory T cells (Treg), by TGF-β in human Vγ9 Vδ2 T cells has been previously reported. Vitamin C is an antioxidant and acts as multiplier of DNA hydroxymethylation. Here we have investigated the effect of the more stable phospho-modified Vitamin C (pVC) on TGF-β-induced FOXP3 expression and the resulting regulatory activity of highly purified human Vγ9 Vδ2 T cells. pVC significantly increased the TGF-β-induced FOXP3 expression and stability and also increased the suppressive activity of Vγ9 Vδ2 T cells. Importantly, pVC induced hypomethylation of the Treg-specific demethylated region (TSDR) in the *FOXP3* gene. Genome-wide methylation analysis by Reduced Representation Bisulfite Sequencing additionally revealed differentially methylated regions in several important genes upon pVC treatment of γδ T cells. While Vitamin C also enhances effector functions of Vγ9 Vδ2 T cells in the absence of TGF-β, our results demonstrate that pVC potently increases the suppressive activity and FOXP3 expression in TGF-β-treated Vγ9 Vδ2 T cells by epigenetic modification of the *FOXP3* gene.

## Introduction

Most T cells express the αβ T-cell receptor (TCR) which serves to recognize peptides presented by MHC/HLA class I or class II molecules to CD8 T cells or CD4 T cells, respectively. The germline TCR repertoire of αβ T cells is highly diverse, due to the large number of available variable (V) Vα and Vβ elements that can be selected during TCR gene rearrangement. By contrast, only a few Vγ and Vδ germline gene elements are available for the recombination of functional human γδ TCR^1^. γδ T cells comprise 2–5% of peripheral blood T cells but are enriched in mucosal tissue. In human peripheral blood, the majority of γδ T cells expresses a Vγ9 Vδ2 TCR, whereas Vδ1 associated with various Vγ elements is predominantly expressed by intestinal γδ T cells^2,3^. Vγ9 Vδ2 T cells recognize pyrophosphate molecules (“phosphoantigens”, pAg) which are intermediates of the eukaryotic mevalonate or the prokaryotic non-mevalonate pathway of isoprenoid biosynthesis^4^. Prototypes of such pAg are the eukaryotic isopentenyl pyrophosphate (IPP) and the prokaryotic (*E*)-4-Hydroxy-3-methyl-but-2-enyl pyrophosphate (HMBPP) which selectively activate Vγ9 Vδ2 T cells at micro- and nanomolar concentrations, respectively^4,5^. Synthetic analogs of naturally occurring pAg like bromohydrin pyrophosphate (BrHPP) have been described, exerting potent and selective stimulation of human Vγ9 Vδ2 T cells^6^. While activation of Vγ9 Vδ2 T cells by pAg does not require HLA class I or class II molecules, there is an indispensable requirement for the transmembrane protein butyrophilin 3 A (BTN3A/CD277)^7^. The current model implies that pAg bind to the intracellular B30.2 domain of BTN3A, thereby initiating a conformational change of the extracellular domain which is then selectively sensed by the Vγ9 Vδ2 TCR^8^.

Activated Vγ9 Vδ2 T cells exert potent and HLA non-restricted cytotoxicity towards many different tumor cells, and therefore have attracted great interest for potential application in cancer immunotherapy^9,10^. However, γδ T cells display a surprising functional plasticity. Depending on the micro-environmental signals, they can be also driven into cytokine producing cells reflecting type 1 (interferon-γ, IFN-γ), type 2 (Il-4, IL-10), type 9 (IL-9)^11^, or type 17 (IL-17, IL-22) patterns^12–14^. Moreover, Vγ9 Vδ2 T cells can acquire the capacity to process exogenous antigen and present it to conventional HLA class I-restricted CD8 T cells (“cross-presentation”), a feature usually restricted to professional antigen-presenting cells like dendritic cells^15,16^. Finally, it has been reported that Vγ9 Vδ2 T cells can also differentiate into suppressive FOXP3^+^ T regulatory (Treg)-like cells when cultured in the presence of TGF-β and IL-15^17^. While suppressive features of Vγ9 Vδ2 T cells are not desirable in the context of anti-tumor activity, such regulatory γδ T cells might be of interest to dampen autoimmunity or allergy^18^.

T-cell activation and differentiation are regulated by epigenetic mechanisms. Several classes of drugs which affect DNA methylation and histone modification have been investigated for their effect on T cells including γδ T cells^19^. Vitamin C (VC) has multiple effects on the immune system, due to its potent anti-oxidant activity, but it also acts as a multiplier of DNA hypomethylation by promoting active Ten-eleven-translocation (Tet) methylcytosine dioxygenases-dependent DNA hydroxymethylation^20,21^. We have recently reported that VC and the more stable and less toxic phospho-modified Vitamin C (pVC) increase the proliferative activity and effector functions of Vγ9 Vδ2 T cells^22^. Moreover, it has been demonstrated that VC promotes the demethylation of the Treg-specific demethylated region (TSDR; also known as conserved non-coding sequence 2, CNS2) located in the *FoxP3* locus in conventional murine CD4 T cells stimulated under Treg-inducing conditions, thereby stabilizing the expression of the Treg-specific master transcription factor FoxP3 and enhancing the regulatory activity of CD4 T cells^23–25^. In this study, we confirm that purified human peripheral blood Vγ9 Vδ2 T cells acquire regulatory activity when activated in the presence of TGF-β. More importantly, we demonstrate that pVC strongly upregulates and stabilizes FOXP3 protein expression, induces hypomethylation in the *FOXP3* TSDR, and increases the suppressive capacity of Vγ9 Vδ2 T cells expanded in the presence of TGF-β. Genome-wide methylation analysis identified additional genes regulated by pVC. We discuss the implications of our findings for the context-dependent modulation of human γδ T-cell functions.

## Materials and Methods

All methods and experiments were carried out in accordance with relevant institutional guidelines and regulations.

### Cell isolation and flow cytometry

Leukocyte concentrates obtained from healthy adult blood donors were provided by the Institute of Transfusion Medicine, UKSH Campus Kiel. Informed consent was obtained from all subjects. This research was performed in accordance with the declaration of Helsinki and was approved by the Ethics Committee of the Medical Faculty of the University of Kiel (Reference D 546/16). Peripheral blood mononuclear cells (PBMC) were isolated by Ficoll-Hypaque (Biochrom, Cambridge, UK) density gradient centrifugation. Total γδ T cells as well as Vδ2 T cells were positively isolated by magnetic cell sorting (MACS) following the manufacturer’s instructions (Miltenyi Biotec, Bergisch-Gladbach, Germany). CD4 T cells were negatively isolated by MACS technology (CD4 T Cell Isolation Kit II, Miltenyi Biotec) followed by the depletion of CD25^+^ Treg using Dynabeads (Life Technologies, Carlsbad, CA, USA). After the use of two consecutives MACS columns (in case of positive selection), the purity of each cell type was typically >97%.

Cells were stained with fluorochrome-conjugated monoclonal antibodies (mAb) directed against CD3 (clone SK7), CD4 (clone SK3) and Ki-67 (clone Ki-67) from Biolegend (San Diego, CA, USA); CD86 (clone FM95) and PD-1 (clone PD1.3.1.3) from Miltenyi Biotec; GITR (clone FAB689P) from R&D Systems (Minneapolis, USA); TCRγδ (clone 11F2), TCR Vδ2 (clone B6), CD103 (clone Ber-ACT8) and FOXP3 (clone 259D/C7) and its appropriate isotype control from BD Biosciences (Heidelberg, Germany); Tet1 (clone GT1462) and its isotype control from ThermoFisher Scientific (Waldham, MA, USA). For intracellular staining of FOXP3, Ki-67 and Tet1, cells were fixed and permeabilized using the FoxP3 transcription factor staining buffer (eBioscience, Thermofisher Scientific) according to the manufacturer´s instructions. Cells were acquired on a LSRII Fortessa cytometer (BD Biosciences) and data were analyzed with FlowJo Software (Tree Star, Ashland, OR, USA)

### Cell culture

Magnetically isolated cells were cultured in 96-well round-bottom plates (Nunc; ThermoFisher Scientific) in medium RPMI 1640 supplemented with 2 mM L-glutamine, 1% penicillin/1% streptomycin, 10 mM HEPES and 10% heat-inactivated fetal bovine serum (complete medium) and incubated at 37 °C in a humidified atmosphere of 5% CO_2_ in air. For the initial γδ T-cell expansion, MACS-purified total γδ (or Vδ2) T cells were stimulated with 300 nM BrHPP (kindly provided by Innate Pharma, Marseille, France) or with Activation/Expander T cell beads (A/E-beads; Miltenyi Biotec). The A/E-beads were coated with 10 µg/mL anti-CD3, 10 µg/mL anti-CD28, and 0.5 µg/mL anti-CD2 mAbs, and were used at 1:1 cells/beads ratio. Cells (50 × 10^3^/well) were cultured for eight days with 50 IU/mL recombinant human IL-2 (Novartis, Basel, Switzerland), 2 ng/mL TGF-β (Peprotech, Hamburg, Germany) in the presence or absence 50 µg/mL (173 µM) phospho-modified Vitamin C (pVC, cat. number A8960; Sigma Aldrich/Merck, Darmstadt, Germany). To test the stability of FOXP3 expression, γδ T cells were expanded for eight days under different conditions as described under Results. Thereafter, cells were washed twice, transferred into new 96-well round-bottom plates and cultured in the presence of 50 IU/mL IL-2 and A/E beads (where indicated) but absence of TGF-β and pVC. After additional six days, cells were analyzed for FOXP3 expression as described above.

### *In vitro* suppression assay

For the *in vitro* suppression assay, γδ T cells were first stimulated for 14 days in the presence of IL-2 and TGF-β and pVC where indicated. On day 14, the expanded γδ T cells (20 × 10^3^/well) were co-cultured for five days with magnetically isolated autologous CD25-depleted CD4 responder T cells (20 × 10^3^/well). The proliferation of CD4 responder T cells and γδ T cells in microculture wells was simultaneously assessed by a previously described flow cytometry-based method, termed standard cell dilution assay (SCDA)^26^. Briefly, expanded cells were harvested, washed and stained for 30 min with FITC-labeled anti-γδ TCR and PE-conjugated anti-CD4 mAb. Shortly before analysis, propidium iodide (PI, 0.2 µg/mL) and a known number of APC-labeled and fixed standard cells were added. Purified CD4 T cells labeled with APC-conjugated anti-HLA class I mAb (clone W6/32) and anti-TCRαβ mAb (clone BMA031), fixed in 1% paraformaldehyde, served as standard cells. The absolute cell numbers of viable γδ T cells and CD4 responder T cells were calculated from the ratios of FITC^+^ γδ T cells/APC^+^ standard cells and PE-CD4^+^ T cells/APC^+^ standard cells, respectively.

*In vitro* suppression capacity was also measured by Carboxyfluorescein succinimidyl ester (CFSE) dilution and analyzed by flow cytometry. To assess the suppression of CD4 responder T cells, negatively isolated CD4 T cells were labeled with CFSE (ThermoFisher Scientific). In brief, CD4 T cells were resuspended at 10^6^ cells/mL, and CFSE was added at a final concentration of 5 µM. Cells were incubated for 5 min at room temperature in the dark, washed twice with PBS/10% FBS, and resuspended in complete RPMI medium. The CFSE-labeled CD4 T cells were stimulated with A/E beads in the absence or presence of 14-day expanded Vδ2 T cells (initially cultured with A/E beads and TGF-β in the presence or absence of pVC). On day five, cells were stained with fixable viability dye (Life Technologies) and anti-CD4 (clone SK3). CD4 responder T cells were gated as live cells (negative for the fixable viability dye), and cell division of the responder cells was assessed by dilution of CFSE. In parallel, at the end of the coculture, FOXP3 stability in the TGF-β-expanded Vδ2 T cells treated or not with pVC, and FOXP3 protein expression in co-cultured CD4 responder T cells were analyzed by flow cytometry.

### DNA methylation analysis

Magnetically isolated Vδ2 γδ T cells were obtained from healthy male donors and were stimulated with BrHPP or with A/E-beads in IL-2 and TGF-β containing complete medium in the presence or absence of pVC. To assess the methylation status of *FOXP3* TSDR, γδ T cells were stained after eight days with anti-FOXP3 mAb and sorted according to their intracellular FOXP3 expression into FOXP3^+^ Vδ2 and FOXP3^−^ Vδ2 T cells. Cell sorting was performed on a FACSAria II cell sorter (BD Biosciences). Genomic DNA was isolated from the sorted cells using the NucleoSpin Tissue kit (Macherey & Nagel, Düren, Germany). An additional step was added to the manufacturer’s protocol to remove formaldehyde-induced crosslinking. Briefly, Chelex-100 beads (Biorad, Hercules, CA, USA) were added after the lysis step and incubated at 95 °C for 15 min in a shaker. The beads were spun down and the supernatant was transferred to a fresh tube. After addition of an adjusted amount of 99.8% ethanol (Merck, Darmstadt, Germany) the following purification steps were performed according to the manufacturer’s protocol. Genomic DNA was converted with bisulfite using the EZ DNA Methylation-Lightning kit (Zymo Research, Irvine, CA, USA) according to the manufacturer’s instructions. The human Treg-specific demethylated region (TSDR) was amplified by PCR using bisulfite-converted DNA, the primers hTSDR-for (5′GAGATGATTTGTTTGGGGGTAGAGGA-3′), hTSDR-rev (5′-bio- AACACCCATATCACCCCACCT-3′) and the ZymoTaq PreMix (Zymo Research) according to the manufacturer’s protocol. The amplificate was sequenced by pyrosequencing using the sequencing primer hTSDR-seq (5′-ATAGTTTTAGATTTGTTTAGATTTT-3′) on a Pyromark Q24 (Qiagen, Hilden, Germany) and analyzed following the manufacturer’s instructions.

For the genome-wide methylation analysis, genomic DNA was extracted from expanded γδ T cells on day eight of the culture using the DNeasy Blood and Tissue Kit (Qiagen, Hilden, Germany), and subjected to Reduced Representation Bisulfite sequencing (RRBS). The isolated DNA was cut by restriction enzyme MspI to produce CpG-rich fragments. End repair process and A-tailing and adapter ligation were performed. Then, DNA was loaded on agarose gel, and 40–220 bp fragments were size selected and subjected to bisulfite treatment using the EZ DNA Methylation-Gold kit (Zymo Research) according to the manufacturer’s instructions. After PCR amplification, the qualified libraries were sequenced using Illumina high-throughput bisulfite sequencing (MethylC-Seq). After sequencing, methylation level was determined by the reads which covered in cytosine (C)^27^, and the methylation level equal to the mC reads number/total C reads number at each reference cytosine as described^28^. The UCSC hg19 annotation was used. Genome segmentation was performed using R package MethylSeekR^29^. Putative differentially methylated regions (DMRs) were identified by comparison of sample1 and sample2 methylomes using windows that contained at least 5 CpG (CHG or CHH) sites with a 2-fold change in methylation level and Fisher test p value <0.05. In addition, we required that both samples should not be hypomethylated in DMR discovery. Two nearby DMRs would be considered interdependent and joined into one continuous DMR if the genomic region from the start of an upstream DMR to the end of a downstream DMR also had 2-fold methylation level differences between sample1 and sample2 with a *p* value <0.05. Otherwise, the two DMRs were viewed as independent. After iteratively merging interdependent DMRs, the final dataset of DMRs was made up of those that were independent from each other.

### Pathway and functional gene ontology (GO) analysis

Genes associated with DMR (gene body and promoter both together) were used as input for the GO analysis using Enrichr, a web-based enrichment analysis tool^30,31^ with human background on default settings. Output term was considered significantly enriched for *p* value ≤ 0.05.

### Statistical analysis

Results of *in vitro* cell culture experiments and FACS data were analyzed with Microsoft Excel 2007. Statistical analysis was performed using Prism 6.01 (GraphPad Software, La Jolla, CA, USA). Statistical significance was calculated with the paired two-tailed Student’s *t* test. Comparative analysis for more than two groups was performed using the Kruskal-Wallis one-way ANOVA with a nonparametric Dunn’s multiple comparisons test. *p* values <0.05 were considered significant and are displayed as * for *p* < 0.05, ** for *p* < 0.01, *** for *p* < 0.001.

## Results

### **Phospho-Vitamin C increases FOXP3 protein-expression in TGF-β-treated γδ T cells**

**Figure 1:**
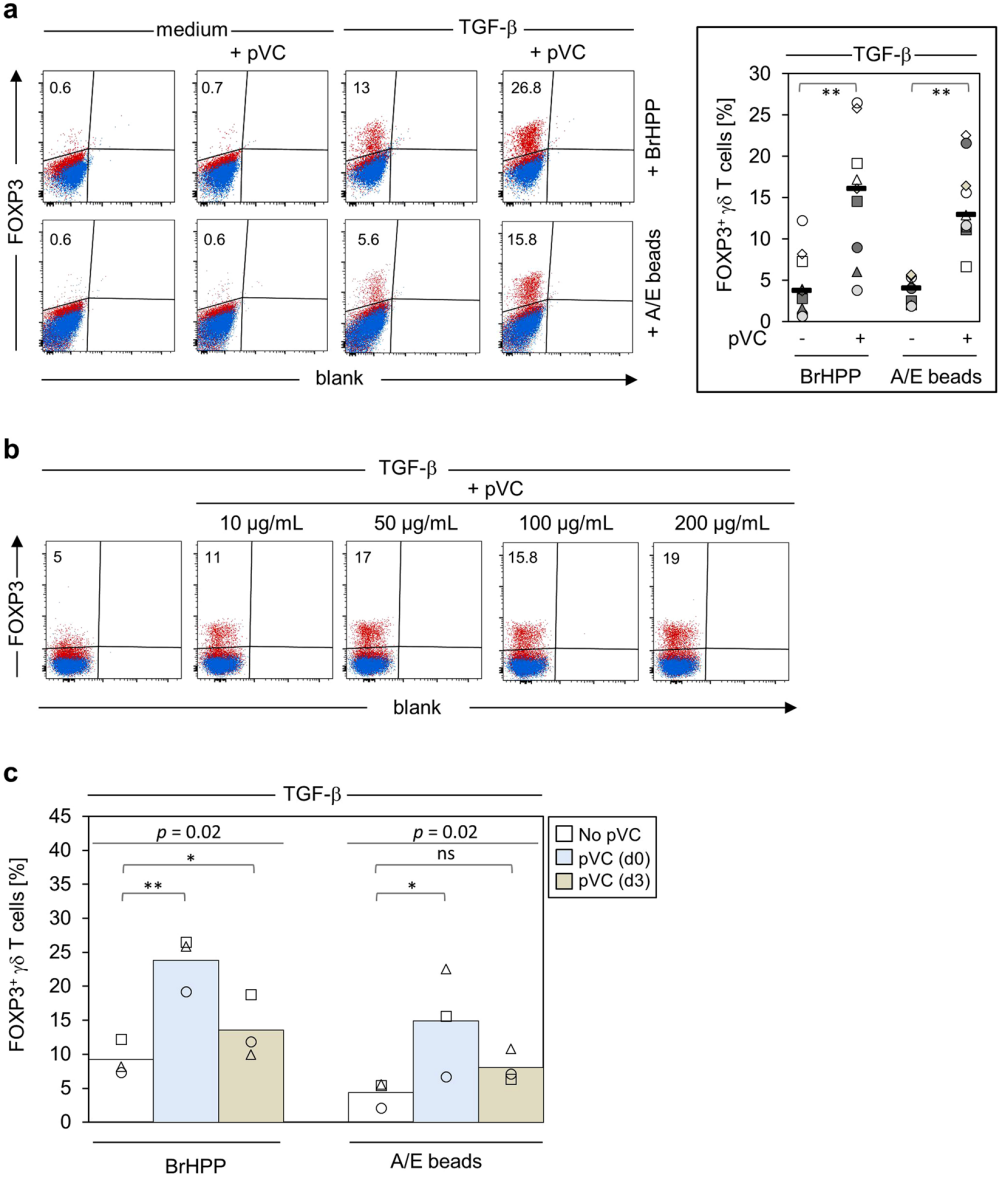
Phospho-Vitamin C enhances FOXP3 expression in human γδ T cells. **(a)** Magnetically isolated γδ T cells were stimulated with BrHPP or A/E-beads (anti-CD2/CD3/CD28 mAb-coated beads) in complete medium supplemented with IL-2 +/− TGF-β in the absence or presence of 50 µg/mL pVC. After eight days, cells were harvested, stained with anti-FOXP3 mAb (red color) and the respective isotype control (blue color). Left: Dot plots of one representative out of nine independent experiments are shown. Numbers indicate the percentage of FOXP3^+^ cells. Right: the graph shows the frequency of FOXP3^+^ γδ T cells in TGF-β-expanded γδ T cells treated or not with pVC. Each symbol represents an individual donor (n = 9). Horizontal bars represent the median. (**b)** Magnetically isolated Vδ2 T cells were stimulated with A/E-beads in complete medium supplemented with IL-2 plus TGF-β under the indicated increasing concentrations of pVC. As in (a), FOXP3 protein-expression was analyzed by flow cytometry on day eight and dot plots of one out of two independent experiments are shown. (**c)** Magnetically isolated γδ T cells were activated with BrHPP or A/E-beads in the presence of IL-2 and TGF-β. Additionally, the cells were left untreated or were treated with pVC (50 µg/mL) at the initiation of cell cultures (d0) or on day three (d3). Each symbol represents an individual donor. The bar chart represents the mean value. Statistical comparison for more than three groups was based on Kruskal-Wallis one-way ANOVA followed by Dunn’s multiple comparisons test. The indicated *p* values refer to the level of significance comparing three groups. *p < 0.05, **p < 0.01 ns, not significant.

**Figure 2:**
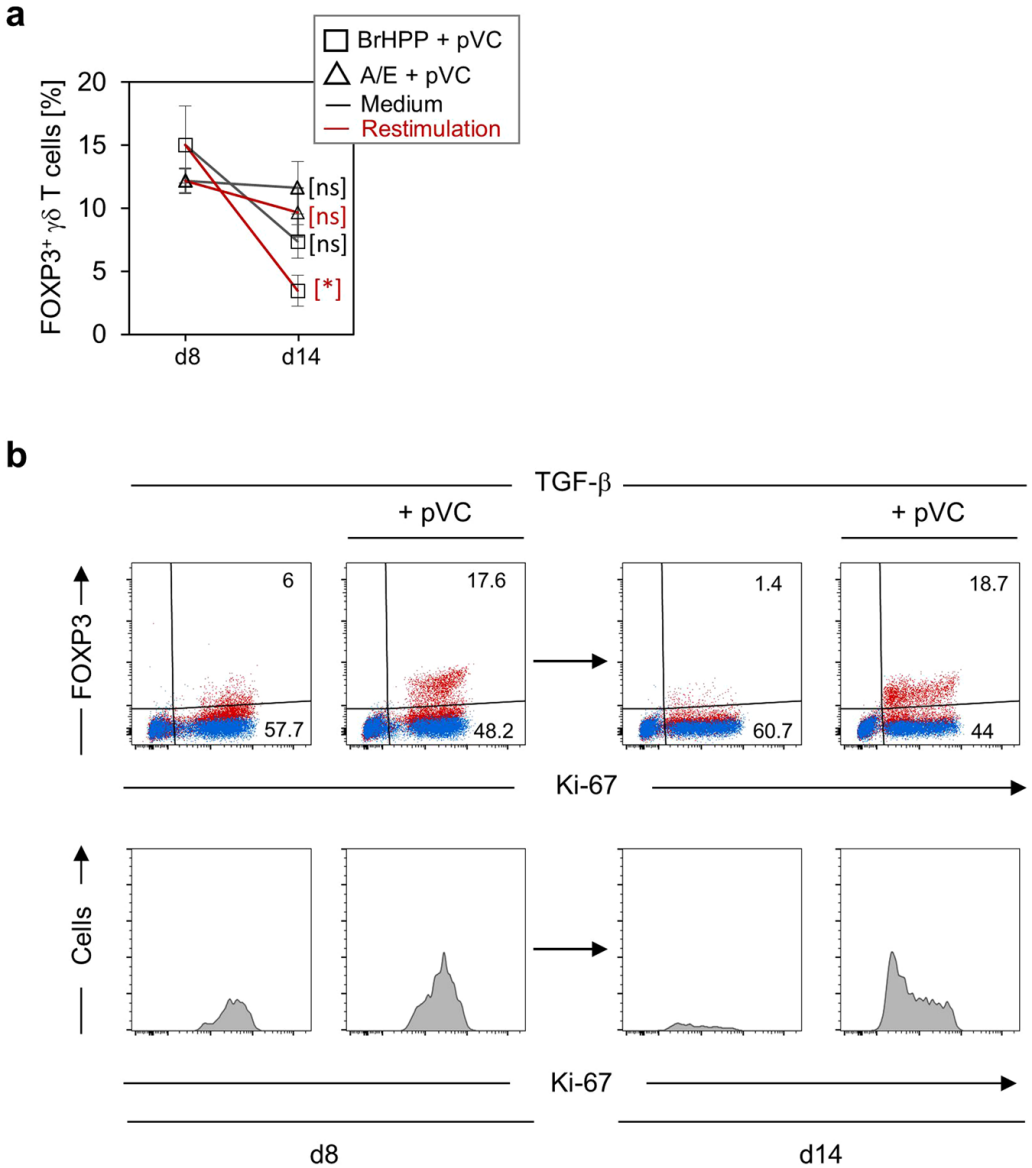
Phospho-Vitamin C induces FOXP3 stability in TGF-β-expanded γδ T cells. **(a)** Magnetically isolated γδ T cells were stimulated with BrHPP (rectangle) or A/E-beads (triangle) in the presence of IL-2 plus TGF-β and additional presence of pVC (50 µg/mL). FOXP3 protein-expression was determined on day eight by flow cytometry. Thereafter, cells were washed and re-seeded in the presence of IL-2 (indicated as Medium, black line) or restimulated with A/E beads in IL-2-containing medium (indicated as Restimulation, red line). FOXP3 protein-expression was again analyzed by flow cytometry after six days (i.e. day 14 after primary activation). The kinetic of FOXP3 protein-expression is depicted as median values ± SEM of six independent experiments. ns, not significant; *p < 0.05 (**b)** Magnetically isolated Vδ2 T cells were stimulated with A/E-beads in complete medium supplemented with IL-2 plus TGF-β and additional presence (or not) of pVC (50 µg/mL). FOXP3 protein-expression was determined on day eight and day 14 by flow cytometry. Upper panel: dot plots of one representative out of three independent experiments depicting FOXP3 protein-expression on Ki-67^+^ Vδ2 T cells. Lower panel: Histograms from the same experiment showing the Ki-67 expression on FOXP3^+^ cells. Numbers indicate the percentage of FOXP3^+^ cells.

Highly purified peripheral blood γδ T cells were stimulated with pAg BrHPP or anti-CD2/CD3/CD28 mAb-coated microbeads (activation/expander beads, A/E-beads) and IL-2 in the presence of different combinations of TGF-β and pVC. After eight days, intracellular FOXP3 expression was determined by flow cytometry. Results of an individual experiment are shown in the left part of Fig. 1a, a summary of several experiments in the right part of Fig. 1a. In the absence of TGF-β, pVC did not induce any FOXP3 expression. In the presence of TGF-β, some FOXP3 protein expression was induced both in BrHPP- and in A/E bead-activated cells which, however, was significantly further increased in cultures supplemented with pVC. Thus, the proportion of FOXP3^+^ γδ T cells (BrHPP: 3.7 ± 3.6%; A/E-beads: 4.0 ± 1.5%) was increased to 16.1 ± 7.5% (BrHPP) and 12.9 ± 4.8% (A/E-beads) upon addition of pVC (Fig. 1a, right part). The presence of pVC also increased the recovery of viable γδ T cells after eight days of culture (Supplemental Fig. S1). Dose titration experiments revealed that the proportion of FOXP3^+^ γδ T cells did not further increase with higher concentrations of pVC (Fig. 1b). Therefore, 50 μg/mL pVC was used throughout all following experiments. Next, we investigated the influence of the time point when pVC had to be added to enhance FOXP3 expression as measured on day eight of *in vitro* expansion. To this end, purified γδ T cells were activated with BrHPP or A/E-beads in the presence of TGF-β, and pVC was added at the beginning of cell culture (d0) or after three days (d3). As shown in Fig. 1c, pVC had to be present at the initiation of the cell culture (d0) to significantly augment FOXP3 protein-expression after eight days. We then asked how efficiently FOXP3 expression was maintained beyond day eight of cell culture. Purified γδ T cells were stimulated with BrHPP or A/E-beads in the presence of TGF-β and pVC. After eight days, cells were analyzed for FOXP3 expression (Fig. 2a, left). The remaining cells were washed and cultured for additional six days in medium supplemented with IL-2 only, or in medium supplemented with IL-2 and A/E beads (Fig. 2a, right). γδ T cells initially activated with A/E beads in the presence of pVC (rectangle in Fig. 2a) maintained their FOXP3 expression upon reanalysis on day 14, whereas BrHPP-activated γδ T cells (triangle in Fig. 2a) tended to lose FOXP3 expression. A similar pattern was observed when the cells were re-challenged with A/E beads on day eight. Under these conditions, γδ T cells initially activated with A/E beads and pVC maintained FOXP3 expression on day 14, in contrast to γδ T cells initially activated with BrHPP which showed significantly less FOXP3 expression on day 14 (Fig. 2a). We also addressed the correlation between cellular proliferation and FOXP3 expression in γδ T cells. To this end, purified γδ T cells were activated with A/E beads and cultured with TGF-β in the absence or presence of pVC. After eight and 14 days, cells were stained for Ki-67 and FOXP3. As shown in Fig. 2b, FOXP3 was expressed on Ki-67-positive cells on day eight and day 14 only in pVC-supplemented cultures. When a gate was set on FOXP3^+^ cells, it became obvious that FOXP3 was expressed on day eight in highly proliferating (i.e., Ki-67^high^) cells, whereas FOXP3 was more expressed in less proliferating (i.e., Ki-67^low^) cells on day 14 (Fig. 2b, lower row).

### γδ T cells expanded with TGF-β and phospho-Vitamin C display potent regulatory activity

**Figure 3:**
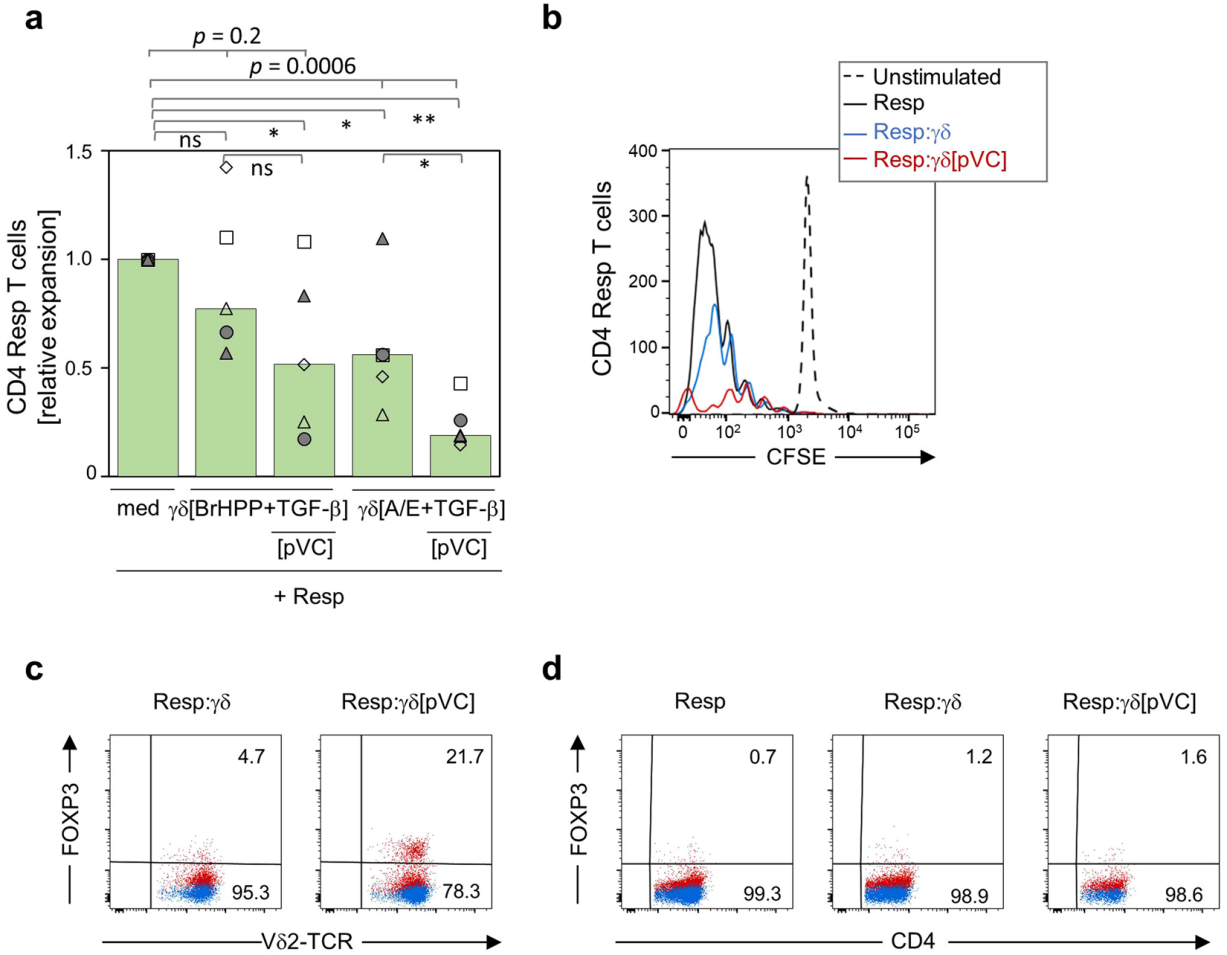
Phospho-Vitamin C enhances the suppressive activity of TGF-β-expanded γδ T cells. MACS-sorted γδ T cells were cultured for 14 days in the presence of TGF-β and IL-2, and in the absence or presence of pVC and BrHPP or A/E-beads as indicated in the figure. Thereafter, autologous CD25-depleted CD4 responder T cells (Resp) were co-cultured with the differentially expanded γδ T cells in the presence of A/E-beads at a responder/γδ ratio of 1:1. (**a)** The number of viable CD4 (responder) per microculture was quantified by the flow cytometry-based SCDA method after five days. The relative expansion of responder CD4 T cells is depicted as a quotient of viable cells *versus* the cell number of CD4 T cells in solo-culture (med) of the respective experiment. (**b)** CFSE-labeled CD4 responder T cells were cultured alone (Resp) or with 14-day TGF-β-expanded Vδ2 T cells (γδ), or with pVC-treated TGF-β-expanded Vδ2 T cells (γδ[pVC]) in the presence of A/E beads. On day 5, the CFSE fluorescence intensity in unstimulated (dashed histogram) and stimulated (black, blue and red histograms) Resp cells was measured by flow cytometry as depicted by the representative overlay histograms (one out of two independent experiments). The proportion of proliferating (CFSE_low_) CD4 T cells was 74% without γδ T cells, 59% with added γδ T cells not expanded with pVC, and 33% with added γδ T cells expanded with pVC. (**c,d)** In parallel to the CFSE measurement on day 5, the FOXP3 protein-expression in (c) TGF-β-expanded Vδ2 T cells treated or not with pVC and (d) the responder cells from the coculture was analyzed by flow cytometry. Dot plots of one representative out of two independent experiments are shown. The indicated *p* values in (a) refer to the statistical significance among three groups and were determined by Kruskal-Wallis one-way ANOVA with Dunn’s multiple comparisons test. *p < 0.05, **p < 0.01, ns not significant; med, medium.

Purified γδ T cells were stimulated with BrHPP or A/E-beads and expanded for 14 days in the presence of TGF-β and additional presence or absence of pVC. Thereafter, γδ T cells were washed and co-cultured with purified autologous CD25-depleted CD4 T cells (“responder T cells”) at 1:1 ratio in the presence of A/E-beads (but absence of exogenous IL-2). After five days, the absolute number of viable CD4 responder T cells and γδ T cells per microculture well was determined by flow cytometry-based SCDA method, which allowed us to simultaneously monitor the expansion of responder CD4 T cells and γδ T cells. When cultured alone, responder CD4 T cells activated with A/E-beads proliferated vigorously (Fig. 3a, “med”; cell number set to 1.0). γδ T cells expanded in the presence of TGF-β reduced the growth of co-cultured CD4 responder T cells, which was more pronounced for A/E-beads-activated γδ T cells. Interestingly, γδ T cells activated in the additional presence of pVC (i.e. [BrHPP+TGF-β + pVC] and [A/E + TGF-β + pVC]) suppressed the CD4 responder T-cell growth more efficiently. Of note, the most potent and significant suppression of CD4 responder T-cell expansion was observed in co-cultures with γδ T cells which had been activated with A/E-beads and cultured for 14 days in the presence of TGF-β and pVC (Fig. 3a). This was confirmed by an alternative *in vitro* suppression assay using CFSE-labeled CD4 responder T cells and γδ T cells expanded for 14 days with A/E beads and TGF-β in the absence or presence of pVC. Again, γδ T cells expanded with pVC were much more suppressive when compared to γδ T cells expanded without pVC (Fig. 3b). We also investigated FOXP3 expression in γδ T cells and in CD4 responder T cells upon co-culture during the suppression assay. As shown in Fig. 3c, FOXP3 expression was maintained in pVC-treated Vδ2 T cells during co-culture with the CD4 responder T cells (Resp:γδ[pVC]), whereas very low FOXP3 expression was observed in the non-pVC-treated co-cultured Vδ2 T cells (Resp:γδ). Importantly, there was no induction of FOXP3 expression in the CD4 responder T cells during the co-culture with Vδ2 T cells (Fig. 3d). During the co-culture with CD4 responder T cells, the number of γδ T cells also increased whenever γδ T cells had been initially activated in the presence of pVC. This was observed both by measuring absolute cell numbers with SCDA as well as by analyzing cell proliferation with the CFSE assay (Supplemental Fig. S2).

To investigate possible effects of pVC on the expression of cell surface markers which might be relevant for the suppressive capacity of γδ T cells or their interaction with CD4 T cells, we analyzed the expression of PD-1, CD86, GITR and CD103 on γδ T cells expanded for eight and 14 days with TGF-β in the absence or presence of pVC. While pVC increased the expression of CD103, it did not modulate the expression of the other analyzed markers (Supplemental Fig. 3).

### **Phospho-Vitamin C induces demethylation of the*FOXP3*TSDR in TGF-β-expanded γδ T cells**

**Figure 4:**
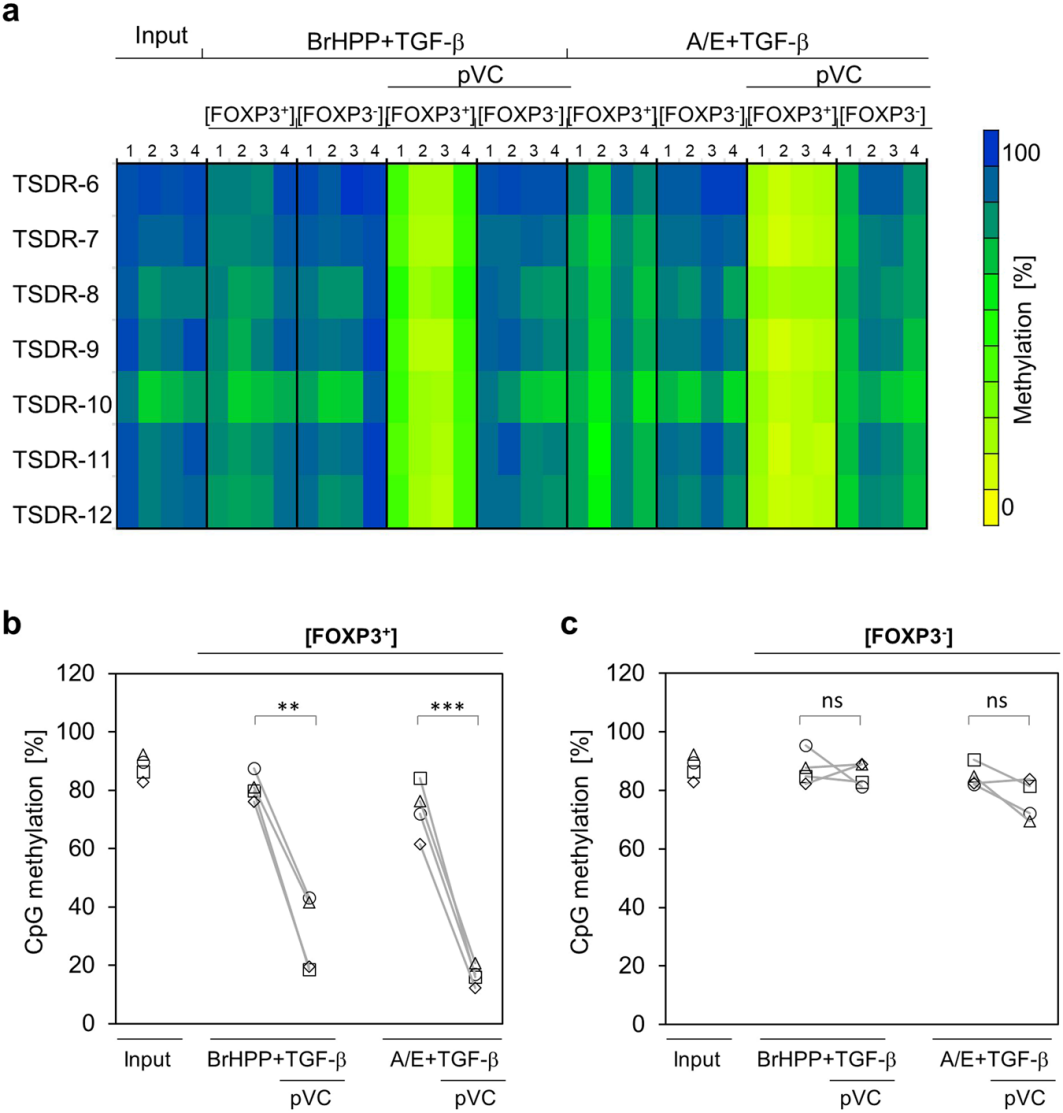
Phospho-Vitamin C induces demethylation of *FOXP3* TSDR in TGF-β-expanded Vδ2 T cells. MACS-sorted Vδ2 T cells were activated with BrHPP or A/E-beads and expanded in complete medium supplemented with IL-2 and TGF-β and the additional presence or absence of pVC (50 µg/mL). On day eight, FOXP3^+^ and FOXP3^−^ Vδ2 T cells were sorted by FACS. Genomic DNA was isolated and subjected to pyrosequencing to determine the methylation status of TSDR. Input cells (MACS-sorted Vδ2 T cells) were included for comparison. (**a)** Data from four independent experiments are depicted. Each row represents the methylation status of an individual CpG motif within the TSDR. The columns show data from independent donors under the indicated experimental conditions. The methylation rates were translated into a color code from yellow (0%) *via* green (50%) up to blue (100%). (**b,c)** Graphs show the average methylation status of the TSDR in (b) FOXP3^+^ and (c) FOXP3^−^ Vδ2 T cells. Each symbol represents an individual donor. **p < 0.01, ***p < 0.001.

Vitamin C acts as a multiplier of DNA hypomethylation by promoting active Tet-dependent DNA hydroxymethylation. Specifically, it has been shown to stabilize Foxp3/FOXP3 expression in murine and human CD4 T cells by inducing demethylation in the *Foxp3*/*FOXP3* TSDR^23–25^. Therefore, we quantified the DNA methylation status of the *FOXP3* TSDR by pyrosequencing. First, we verified that freshly isolated human γδ T cells express Tet proteins. γδ T cells were isolated by negative selection and stained intracellularly with anti-Tet1 antibody. 74 and 85% of γδ T cells expressed Tet1 in two independent experiments (Supplemental Fig. S4). Next, purified γδ T cells were activated with BrHPP or A/E-beads and cultured with IL-2 in the presence of different combinations of TGF-β and pVC. After eight days, cells were sorted into FOXP3^+^ and FOXP3^−^ populations and the DNA methylation of the *FOXP3* TSDR was analyzed. The purity of sorted FOXP3^+^ and FOXP3^−^ γδ T-cell populations from a representative experiment is shown in Supplemental Fig. S5. As illustrated in the heatmap in Fig. 4a, FOXP3^+^ cells cultured in the absence of pVC contained highly methylated TSDRs. Strikingly, FOXP3^+^ γδ T cells activated with BrHPP or A/E-beads in the presence of pVC revealed a strong demethylation in the analyzed TSDRs (Fig. 4a,b), whereas FOXP3^−^ γδ T cells maintained methylated TSDRs irrespective of pVC supplementation (Fig. 4a,c). A statistical analysis of the average degree of methylation of seven CpG sites within the *FOXP3* TSDR in FOXP3^+^ and FOXP3^−^ γδ T cells is presented in Supplementary Fig. S6. Taken together, these results clearly demonstrate that pVC specifically induces *FOXP3* TSDR demethylation in FOXP3^+^ but not FOXP3^−^ γδ T cells.

### Global changes in DNA methylation support stability of phospho-Vitamin C-induced FOXP3 expression in TGF-β expanded γδ T cells

**Figure 5:**
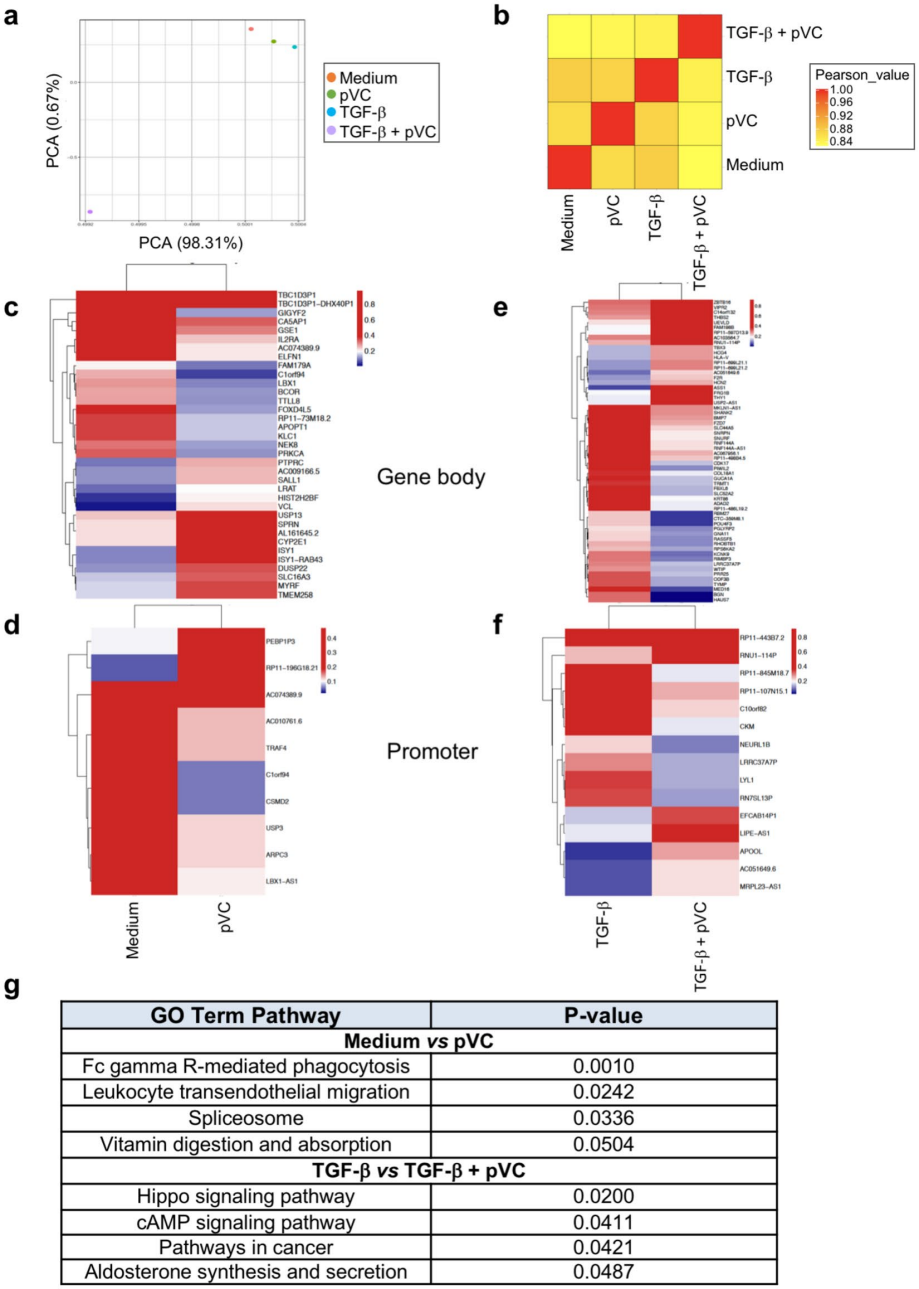
Global DNA methylation analysis of γδ T cells under various conditions using Reduced Representation Bisulfite Sequencing (RRBS). Purified Vδ2 T cells were expanded for eight days in the presence or absence of TGF-β and/or pVC and RRBS analysis was performed. (**a)** The principal component analysis (PCA) was performed on all mCG sites obtained from the RRBS experiment. The analysis of differentially methylated regions (DMRs) was performed as described in the methods section. (**b)** The Pearson’s correlation analysis was performed on the sample groups as shown in the figure. (**c)** The gene body and **(d)** promoters of the respective genes found in DMRs are shown for the comparison between [medium control] and [pVC] treatment, while **(e,f)** show the same analysis for the comparison of [TGF-β] and [TGF-β + pVC]. (**g)** The functional gene ontology analysis was done by using Enrichr web-based tool. Table summarizes enriched pathways and respective p-value for the genes associated with DMRs obtained from the comparison of [medium control] and [pVC], and between [TGF-β] and [TGF-β + pVC]. The statistically significant (p-value ≤ 0.05) pathways are presented only for enrichment analysis.

Since pVC specifically induces *FOXP3* demethylation and stability of FOXP3 expression, we further explored the effect of pVC on TGF-β expanded γδ T cells. To this end, we performed genome-wide DNA methylation analysis using Reduced Representation Bisulfite Sequencing (RRBS) method within the same experimental set-up, i.e. comparing γδ T cells activated for eight days with BrHPP in the absence or presence of TGF-β and/or pVC. The effect of pVC and TGF-β treatment on γδ T cells can be observed in the principal component analysis (PCA), separating samples based on the treatment condition (Fig. 5a). Strikingly, a clear separation of the [pVC + TGF-β]-treated γδ T cells in comparison to the other conditions was observed. The correlation analysis using Pearson’s method showed closer correlation between only [pVC] and only [TGF-β], while [TGF-β + pVC] was least correlated (Fig. 5b). Hence, PCA and correlation analysis revealed global changes in the DNA methylation levels. However, various sequence context (mCG, mCHG, mCHH) of genomic regions such as promoter and CpG islands (CGI) (Supplemental Fig. S7a), and also the genome segmentation (Supplemental Fig. S7b) in partially methylated domains (PMDs) showed slight changes due to +/−TGF-β and +/−pVC conditions. As validated above for its functional role, *FOXP3* was found in the fully-methylated regions (FMR) by genome segmentation using MethylSeekR (data not shown). In line with the above-mentioned observations, pVC-induced *FOXP3* demethylation in TGF-β-expanded γδ T cells is represented using UCSC track viewer (Supplemental Fig. S8). Next, we performed differentially methylated region (DMR) analysis for [medium control] *versus* [pVC] and for [TGF-β] *versus* [TGF-β + pVC] groups to evaluate the pVC-induced regulation of genes/promoters in γδ T cells (Supplementary Table 1). Comparing medium control and pVC, functionally important DMRs contained genes such as *IL2RA*, *BCOR, PTPRC, TRAF4, PRKCA* which were found in the respective genes body and promoters (Fig. 5c,d, Supplementary Fig. S9). The DMR analysis investigating an effect of pVC on TGF-β-expanded γδ T cells revealed gene body or promoters associated with genes such as *FZD7*, *ZBTB16* and *MED16* (Fig. 5e,f, Supplemental Fig. S10). Additionally, to search for different functional features, we performed gene ontology (GO) analysis of genes contributing to DMR. Interestingly, GO terms showing statistically significant enrichment revealed an involvement of the vitamin digestion and absorption pathway for [medium control] *versus* [pVC] (Supplementary Table 2; Fig. 5g), and Hippo and cAMP signaling pathways for [TGF-β] *versus* [TGF-β + pVC] (Supplementary Table 3; Fig. 5g). Overall, the RRBS analysis may support our previous report on stimulatory effects of pVC on γδ T-cell proliferation and effector functions in the absence of TGF-β^22^. In addition, the RRBS analysis revealed an effect of pVC on TGF-β-treated γδ T cells at the level of gene regulation which may add a regulatory component to the *FOXP3* demethylation and cellular functional properties, consistent with our functional results.

## Discussion

Vitamin C is an essential micronutrient with pleiotropic functions in the human organism. It is an antioxidant and free radical scavenger and is an indispensable cofactor for many enzymatic reactions^32^. VC also has multiple effects on the immune system. It has been shown to stimulate T-cell differentiation from early progenitors and to promote the differentiation from CD4^−^CD8^−^ double-negative precursors to the CD4^+^CD8^+^ double-positive stage^32^. Furthermore, VC also enhances the proliferation and cytokine production of mature T cells^33,34^. Moreover, it plays an important role in remodeling the epigenome by increasing the activity of Tet proteins and Jumonij-C domain-containing histone demethylase (JHDMs)^35^. The epigenetic remodeling activities of VC stabilize Foxp3/FOXP3 expression in murine and human Treg cells^23^ and regulate IL-17 production^36^. We have recently investigated the effects of VC and pVC on the *in vitro* activation and effector functions of human γδ T cells. We found that VC and pVC significantly enhanced their proliferative and metabolic activity as well as their cytokine production^22^. We also observed that γδ T cells expanded in the presence of pVC exerted stronger cytotoxic capacity. In this study, we performed a genome-wide methylation analysis comparing γδ T cells expanded in the presence or absence of pVC. We identified several genes potentially contributing to the increased growth and effector function of γδ T cells activated in the presence of pVC, including *IL2RA* and *BCOR*, a co-repressor of the BCL6 repressor protein with a role in regulating T-cell fate decision^37,38^. It is well established that large numbers of Vγ9 Vδ2 T cells with cytotoxic activity against a wide variety of leukemias, lymphomas and solid tumors can be easily generated from PBMC by stimulation with zoledronic acid (which induces the endogenous accumulation of IPP) and IL-2^39–41^. The exogenous supply of VC or pVC might thus help to improve the *in vitro* expansion and effector functions of γδ T cells and thereby to overcome the so far limited success of γδ T-cell immunotherapy^42^.

However, human γδ T cells can also acquire suppressive activity associated with significant FOXP3 expression when activated in the presence of TGF-β^17^. Such FOXP3^+^ γδ T cells were able to suppress CD4 T-cell proliferation, which would be an unwanted activity of γδ T cells applied in adoptive cell therapy. Strikingly, however, we previously observed that TGF-β can also enhance the cytotoxic effector function when purified γδ T cells are expanded in the presence of TGF-β^43^. In the present study, we therefore analyzed in detail the effects of a combined exposure to TGF-β and pVC on Vγ9 Vδ2 T cells. We observed that addition of pVC significantly increased FOXP3 expression in purified Vγ9 Vδ2 T cells activated with pAg BrHPP or A/E-beads in the presence (but not absence) of TGF-β. We also observed that γδ T cells cultured with pVC (particularly those initially activated with A/E-beads) maintained their FOXP3 expression for at least 14 days, and more potently inhibited the proliferation of co-cultured CD4 responder T cells. The suppressive capacity of Vδ2 T cells expanded in the presence of TGF-β plus pVC was verified by two independent experimental approaches, i.e. the measurement of absolute numbers of viable CD4 T cells by flow cytometry as well as the widely used CFSE dilution assay. While FOXP3 expression was high on proliferating γδ T cells (as revealed by high Ki-67 expression) after eight days of stimulation, FOXP3 expression was maintained in Ki-67^low^ cells after 14 days, indicating that the pVC-effect on FOXP3 expression is preserved also when γδ T cells reduce their proliferative activity.

For the first time, we could demonstrate that pVC induced the demethylation of *FOXP3* TSDR in human γδ T cells, as revealed by analyzing sorted FOXP3^+^ and FOXP3^−^ cells from γδ T-cell cultures supplemented with TGF-β and pVC. These results are well in line with previous reports on the induction of *FOXP3* TSDR demethylation and stability by VC in murine CD4 T cells^23,24,44^. Remarkably, however, such *FOXP3* TSDR demethylation was only observed in FOXP3^+^ γδ T cells sorted from [TGF-β + pVC]-supplemented cultures and not in FOXP3^+^ sorted γδ T cells derived from cultures with TGF-β but without pVC (Fig. 4, Supplemental Fig. S6). We conclude that transient and low level FOXP3 protein expression can be induced in human γδ T cells by TGF-β alone, but demethylation of *FOXP3* TSDR required for stable FOXP3 expression (a prerequisite for stable suppressive activity of Treg^45,46^) only occurs after addition of pVC. Furthermore, we observed that pVC increased FOXP3 protein-expression when added together with the TCR stimulation and not at later time point. This might suggest that pVC modulates TCR-induced signaling implicated in the process of FOXP3 induction. Additional investigations are required, however, to identify the target(s) of pVC in the TCR signaling involved in the induction of FOXP3. After removal of the remaining TGF-β and pVC on day eight, γδ T cells initially stimulated with A/E-beads (but not BrHPP) and pVC maintained their FOXP3 protein expression for at least another six days. This may suggest an important role for CD28 co-stimulation (as delivered by A/E beads stimulation) in the pVC-mediated maintenance of FOXP3 in TGF-β-expanded γδ T cells. Such a role for CD28 co-stimulation has been reported for TGF-β-induced thymic regulatory T cells^47^. The more potent regulatory activity of A/E-beads- as compared to BrHPP-expanded γδ T cells is well in accordance with the extended maintenance of FOXP3 expression in the A/E-beads-activated γδ T cells.

In view of the striking effects of pVC on the *FOXP3* TSDR demethylation in TGF-β-treated γδ T cells, we also performed genome-wide DNA methylation analysis by RRBS. The DMR analysis revealed gene body or promoters associated with several genes with possible relevance for regulatory activity such as *FZD7* and *ZBTB16*. Both are key transcription factors with possible relevance for Treg-cell lineage determination and function^48–50^. Furthermore, our pathway analysis and functional gene ontology (GO) analysis of genes contributing to DMR revealed an involvement of the Hippo^51^ and cAMP signaling pathways for [TGF-β] *versus* [TGF-β + pVC]-supplemented γδ T-cell cultures. Both Hippo as well as cAMP pathways are known to play important roles in the regulation of Treg activity^52,53^. In view of the recent characterization of inflammatory Treg based on the involvement of the Wnt-signaling pathway, regulatory genes like *FZD7* and *ZBTB16* might contribute to the increased cytotoxic potential of TGF-β-expanded γδ T cells, which we have recently published^43^. Taken together, the results obtained from the RRBS analysis of TGF-β-treated γδ T cells indicate that pVC affects the methylation level of a number of genes in addition to *FOXP3*, which might be also important for the regulatory activity of γδ T cells.

The present results together with our previous studies on the effects of TGF-β and pVC on the *in vitro* differentiation of human peripheral blood γδ T cells provide important insights into the regulation of plasticity of human γδ T cells. Furthermore, our study has implications for the optimization of γδ T-cell based immunotherapy, notably for the adoptive transfer of *in vitro* expanded γδ T cells. When starting with purified γδ T cells (i.e., not with PBMC where TGF-β has a negative impact on the proliferative activity), both TGF-β and VC/pVC, when added independently, can actually increase the expansion and functional activity as measured by cytokine production and cytotoxic effector activity^11,22,43^. However, in combination, TGF-β and pVC increase the regulatory activity of Vγ9 Vδ2 T cells by enhancing and stabilizing FOXP3 protein expression as a consequence of the demethylation of the *FOXP3* TSDR, which is a known pre-requisite for functionally active Tregs^54^. The functional significance of the additional differentially methylated genes as revealed by RRBS requires further investigation.

Treg cells are crucial for the maintenance of immune homeostasis and the prevention of autoimmune and inflammatory disorders^55–58^. The importance of alloantigen-specific Treg in the context of clinical transplant tolerance was recently highlighted. A superior immunosuppressive capacity of these cells was reported^59^. Interestingly, a previous study demonstrated that the addition of VC to alloantigen-specific αβ Treg cultures led to the generation of a stable Treg population (epigenetically akin to naturally occurring Treg) with enhanced ability to promote skin allograft acceptance^44^. Although some studies have highlighted the potential involvement of regulatory γδ T cells in the pathogenesis of some autoimmune diseases^60–62^, others provided evidence that γδ T cells could exert regulatory functions resulting in the prevention of autoimmune disorders^63,64^. In line, it was also found that, regulatory γδ T cells induced in the presence of decitabine potently suppressed graft-versus-host-disease *in vivo*^65^. Our present study demonstrates that the addition of pVC to TGF-β-stimulated γδ T-cell cultures facilitates the induction of immunosuppressive cells. It remains to be investigated if the FOXP3^−^positive γδ T cells generated by combined TGF-β and pVC treatment, are superior suppressive cells in comparison to conventional Treg cells in certain situations, due to their homogeneous expression of the pAg-reactive Vγ9 Vδ2 TCR.

## Supplementary information

Supplemental Figures.

Table S1.

Table S2.

Table S3.
