# Supplementary material for: Vitamin C supports conversion of human γδ T cells into FOXP3-expressing regulatory cells by epigenetic regulation
Source: Sci Rep. 2020 Apr 16;10:6550. doi: 10.1038/s41598-020-63572-w (PMC7162875; doi:10.1038/s41598-020-63572-w)
Supplement: Supplementary file 1 — Supplemental Figures. [file 41598_2020_63572_MOESM1_ESM.pdf]

Vitamin C supports conversion of human γδ T cells into FoxP3-expressing regulatory cells by epigenetic regulation

Léonce Kouakanou[1], Christian Peters[1], Qiwei Sun[2], Stefan Floess[3], Jaydeep Bhat[1,4], Jochen Huehn[3], Dieter Kabelitz[1*]

Supplemental Fig. S1: Phospho-Vitamin C enhances the proliferation of TGF-β-expanded γδ T cells. Magnetically isolated γδ T cells were stimulated with BrHPP or A/E-beads in complete medium supplemented with IL-2 +/- TGF-β in the absence or presence of 50 µg/mL pVC. At day eight, the number of viable cells was determined by microscopical inspection (with dead cells excluded by eosin staining). Each symbol represents an individual donor. The bar chart represents the mean value. $^{**}p < 0.01$

Supplemental Fig. S2: Absolute cell numbers of the proliferating pVC-treated (or not) expanded γδ T cells after co-culture with CD4 responder T cells. MACS-sorted γδ T cells were cultured for 14 days in the presence of TGF-β and IL-2, and in the absence or presence of pVC and BrHPP or A/E-beads as indicated in the figure. Thereafter, autologous CD25-depleted CD4 responder T cells (Resp) were co-cultured with the differentially expanded γδ T cells in the presence of A/E-beads at a responder/γδ ratio of 1:1. (**a)** The number of viable γδ T cells per microculture was quantified after five days by the flow cytometry based SCDA method. The relative expansion of γδ T cells is depicted as a quotient of viable cells *versus* the cell number of CD4 T cells in solo-culture (med) of the respective experiment. The bar chart represents the median value**. (b)** Resp were cultured for five days in the presence of A/E beads with TGF-β-expanded Vδ2 T cells (γδ) and pVC-treated TGF-β-expanded Vδ2 T cells (γδ[pVC]) both labeled with CFSE dye. Representative histograms of one out of two experiments show the CFSE dilution profile of stimulated (red shaded histogram) and unstimulated (gray shaded histogram) Vδ2 T cells.

Supplemental Fig. S3: Effect of phospho-Vitamin C on the surface marker expression of TGF-β-expanded γδ T cells. Magnetically isolated Vδ2 T cells were stimulated with A/E-beads in complete medium supplemented with IL-2 plus TGF-β and additional presence (or

not) of pVC (50 µg/mL). On day eight and day 14 after primary stimulation, cell surface expression of (**a)** CD86 (clone FM95), (**b)** PD-1 (clone PD1.3.1.3), (**c)** GITR (clone FAB689P) and (**d)** CD103 (clone Ber-ACT8) was measured by flow cytometry. Dot plots of one representative out of three independent experiments are shown. Numbers indicate the percentage of positive cells.

Supplemental Fig. S4: Expression of Tet-1 in freshly isolated human γδ T cells. Negatively isolated γδ T cells were stained with anti-Tet1 mAb (red color) and the respective isotype control (blue color). Dot plots of two independent experiments from two donors are shown. Numbers indicated the percentage of positive cells.

Supplemental Fig. S5: Purity of sorted FOXP3$^+$ and FOXP3$^-$ subpopulations from the TGF-β/pVC-expanded Vδ2 T cells. Purified Vδ2 T cells were activated with BrHPP or A/E beads and cultured in the presence of IL-2 and different combinations of TGF-β and pVC as indicated. After eight days, cells were sorted into FOXP3$^+$ and FOXP3$^-$ subpopulations. Dot plots of one representative experiment are shown. Numbers indicate the percentage of FOXP3$^+$ cells among the expanded Vδ2 T cells (pre-sort), and the sorted FOXP3$^-$ and FOXP3$^+$ cell populations.

Supplemental Fig. S6: Quantification of *FOXP3* TSDR methylation levels upon phospho-Vitamin C treatment. (**a), (b)** Graphs show the average methylation of seven CpG sites within the *FOXP3* TSDR in (a) FOXP3$^+$ fraction and (b) FOXP3$^-$ fraction. Left panel: [BrHPP + TGF-β]-expanded Vδ2 T cells, right panel; [A/E + TGF-β]-expanded Vδ2 T cells. Each symbol represents an individual donor. The bar chart represents the median value. **$p < 0.01$, ***$p < 0.001$, ns not significant

Supplemental Fig. S7: RRBS-based distribution of methylated cytosine found in various sequence context and genomic segments. (a) The percentage of methylated Cytosine in different sequence context for different treatment conditions is presented separately for promoter (upper panel) and CpG islands (CGI; lower panel): CG, CHG, CHH (H = A, T, or C). Each symbol represents an individual donor. The bar chart represents the mean value. **(b)** The genome segmentation was performed using R-package MethylSeekR and the weighted average methylation levels within gene body and promoters were plotted for various

experimental conditions. Abbreviations used: FMR, fully methylated regions; LMR, low methylated regions; PMDs, partially methylated domains; UMR, unmethylated regions.

Supplemental Figs. S8, S9, S10: UCSC browser screenshots of different genes. Within the same experimental conditions, the peak coverage for genes are represented in Fig. S8 for *FOXP3* (chrX:491,138,84-491,142,25) found in FMR; and DMR-associated genes in Fig. S9 for IL2RA (upper track; chr10:609,592,0-609,625,0) and *BCOR* (lower track; chr10:609,592,0-609,625,0), in Fig. S10 for *FZD7* (upper track; chr2:202,900,170-202,900,465) and *ZBTB16* (lower track; chr11:113,962,568-113,962,780). Note that the sites with coverages below 4 were removed from the analysis and representation. The coverage in *FOXP3* gene of the pVC only treatment group is below 4.

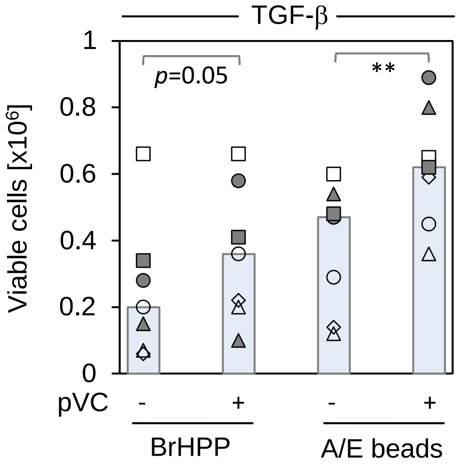

Supplemental Figure S1

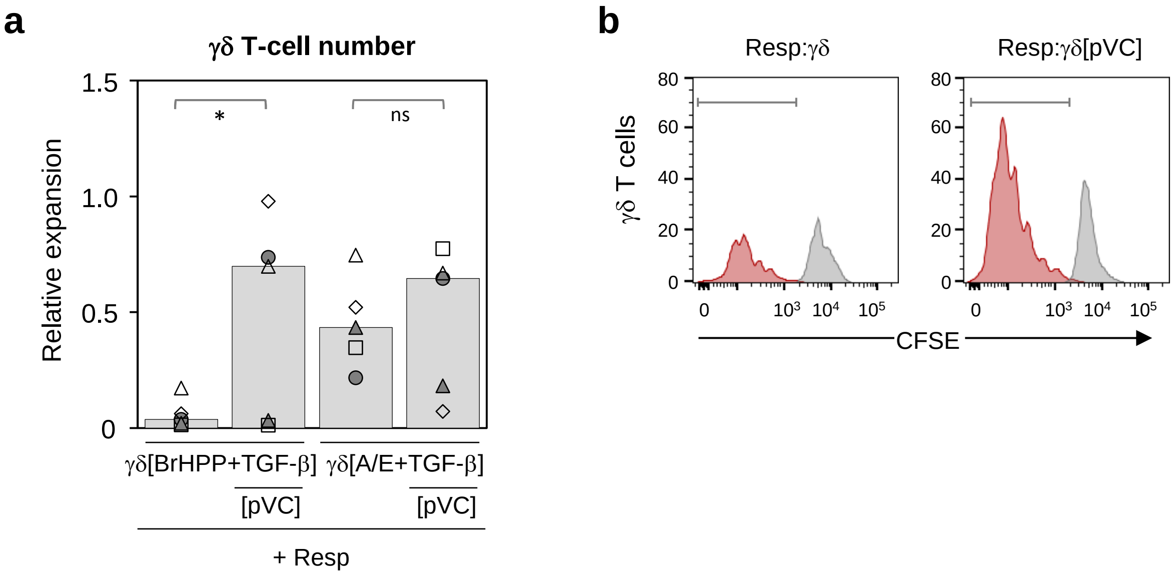

Supplemental Figure S2

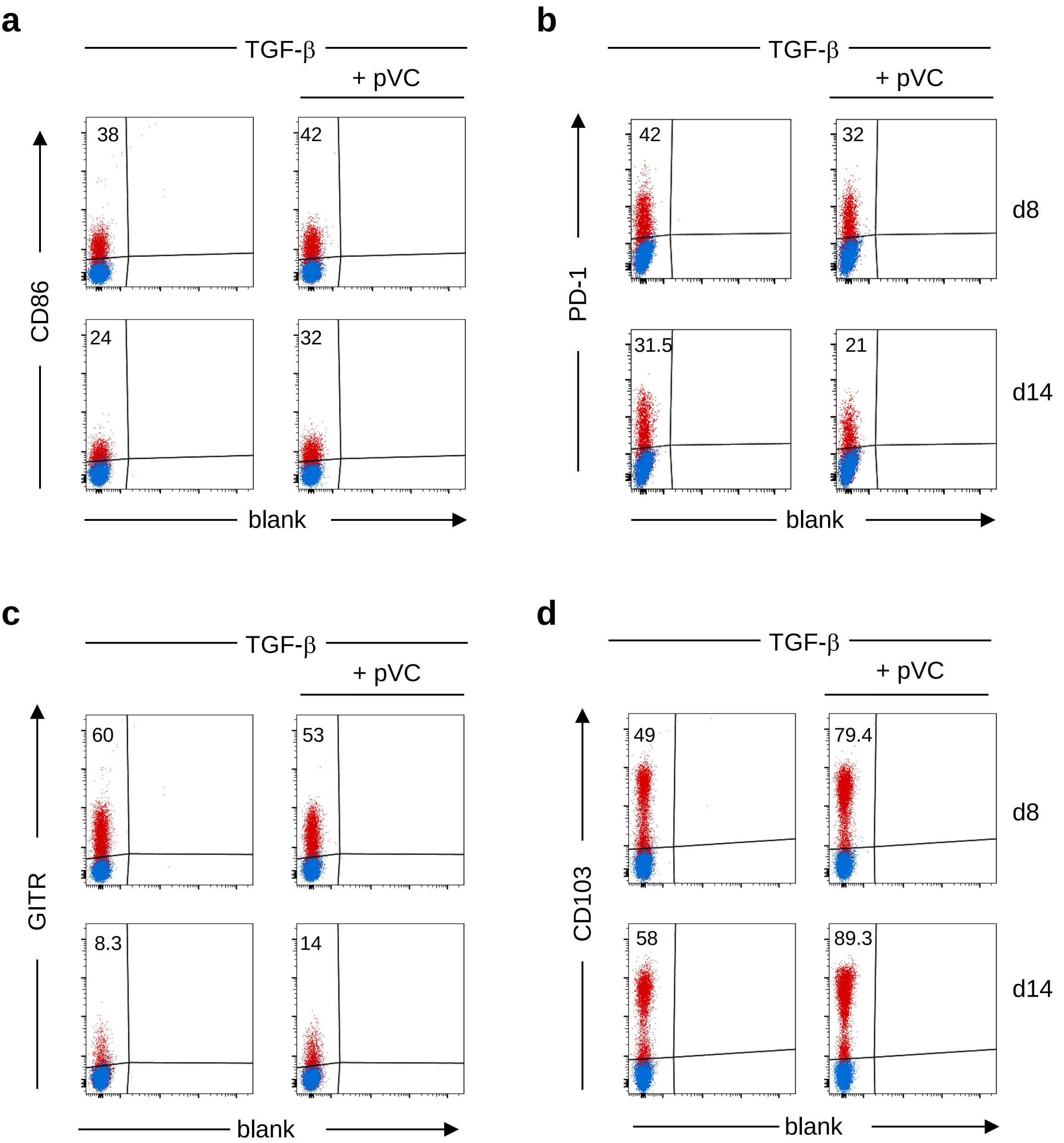

Supplemental Figure S3

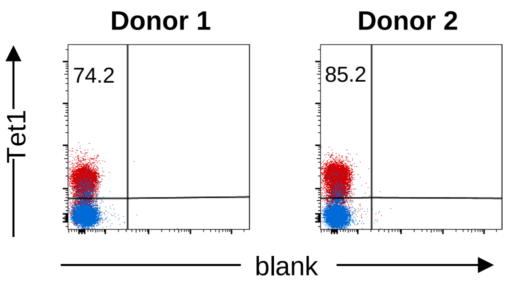

Supplemental Figure S4

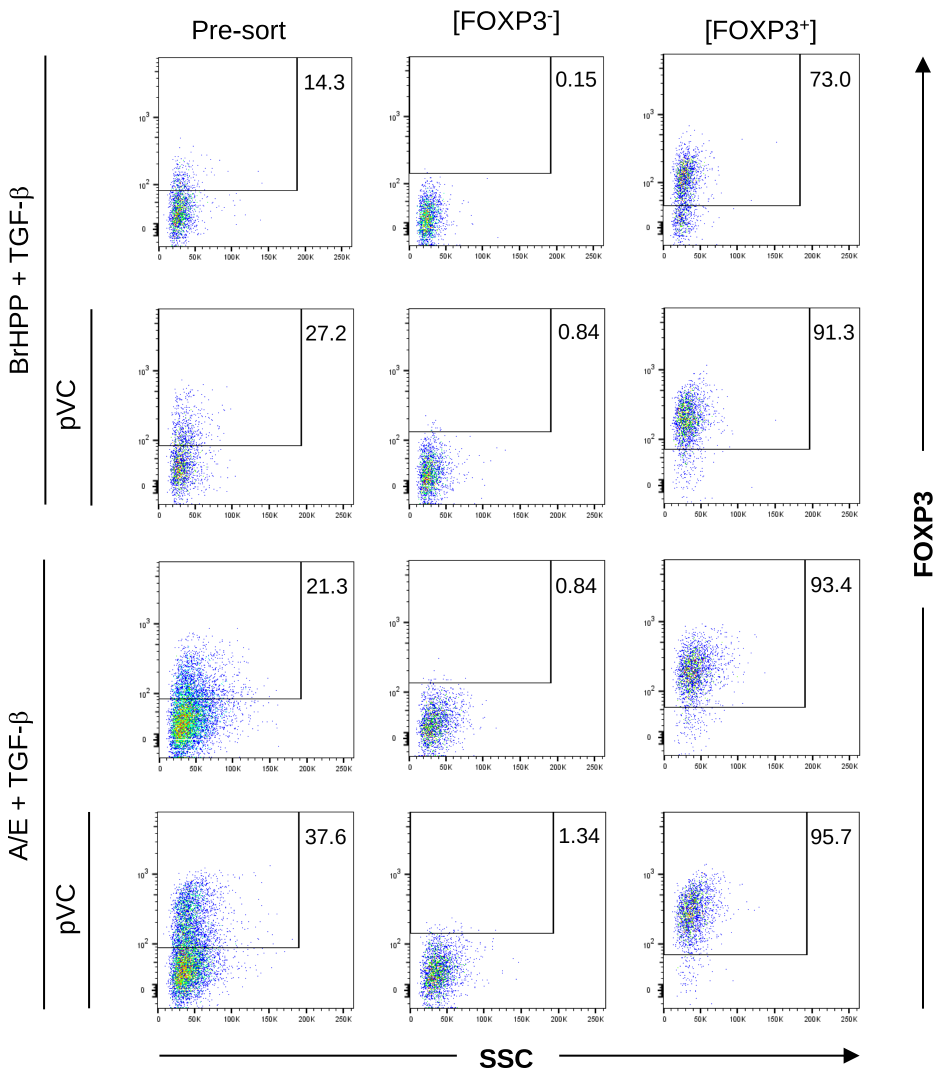

Supplemental Figure S5

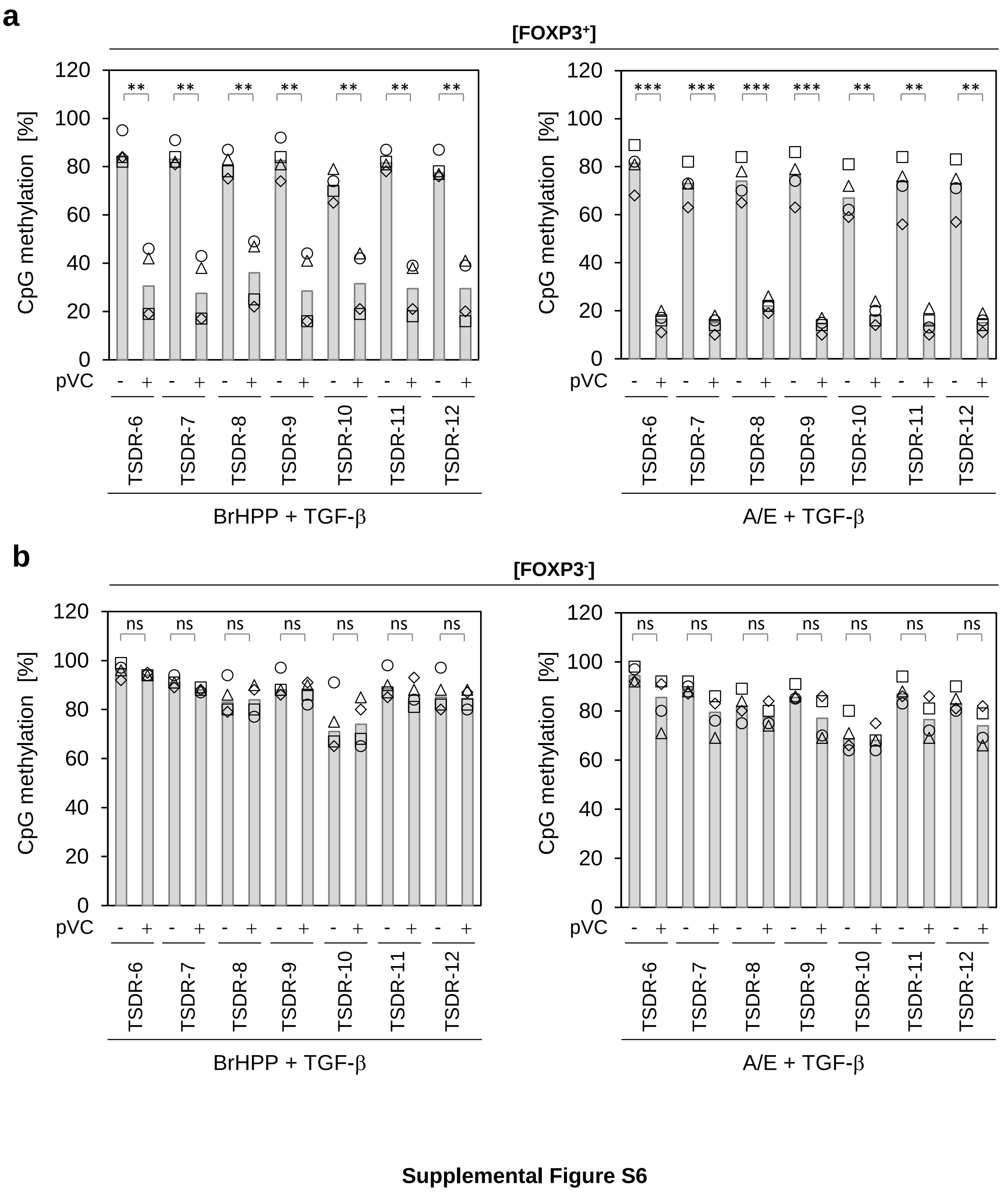

Supplemental Figure S6

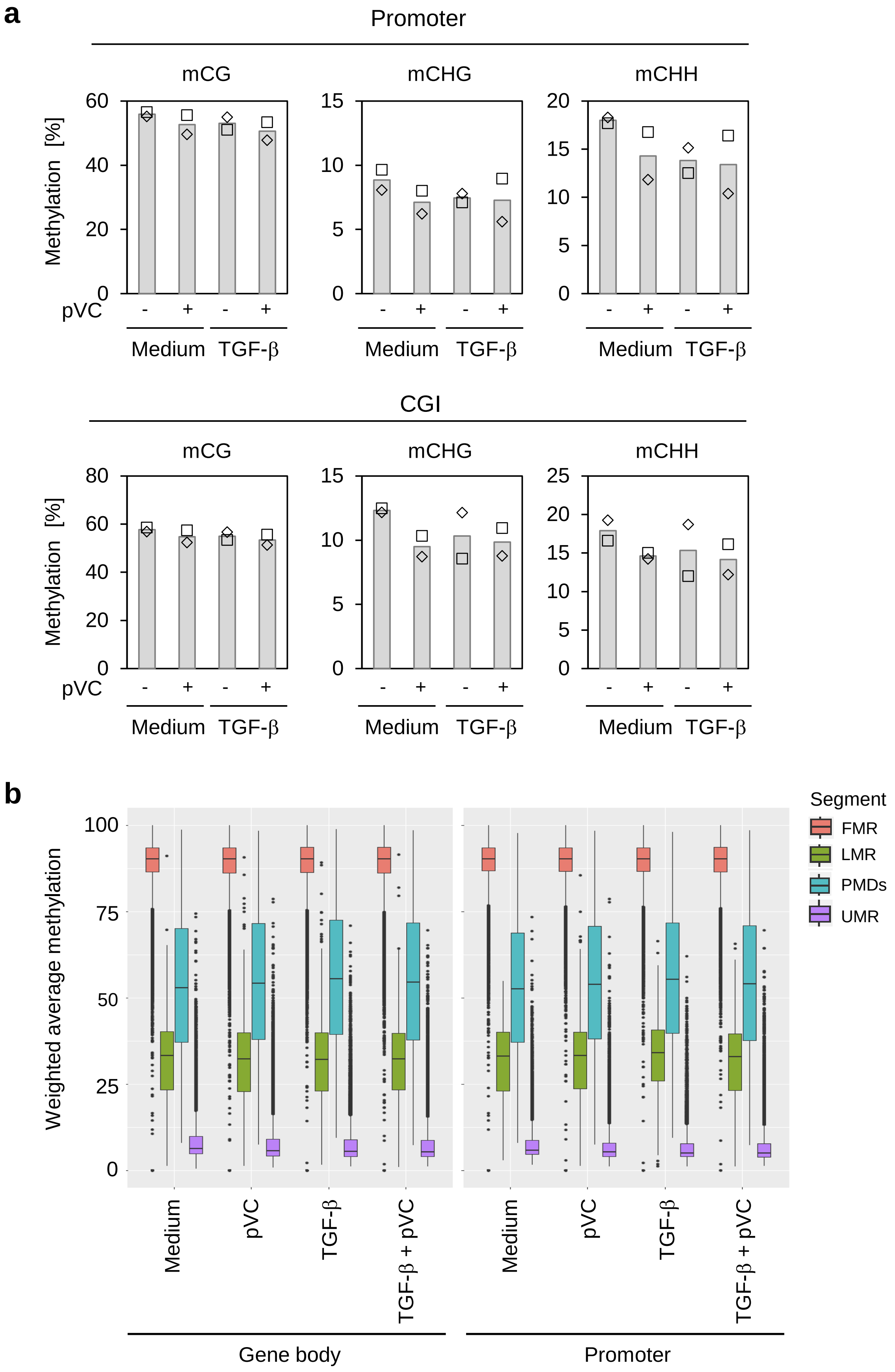

Supplemental Figure S7

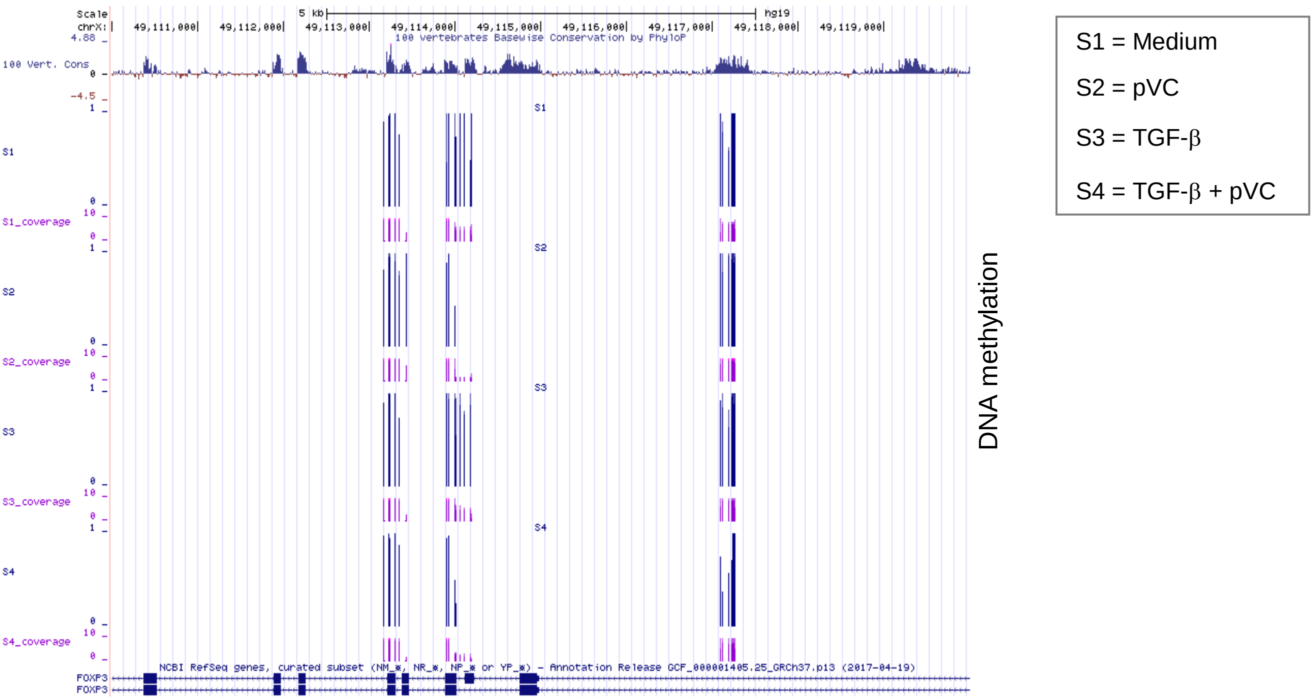

Supplemental Figure S8

S1 = Medium

S2 = pVC

S3 = TGF-β

S4 = TGF-β + pVC

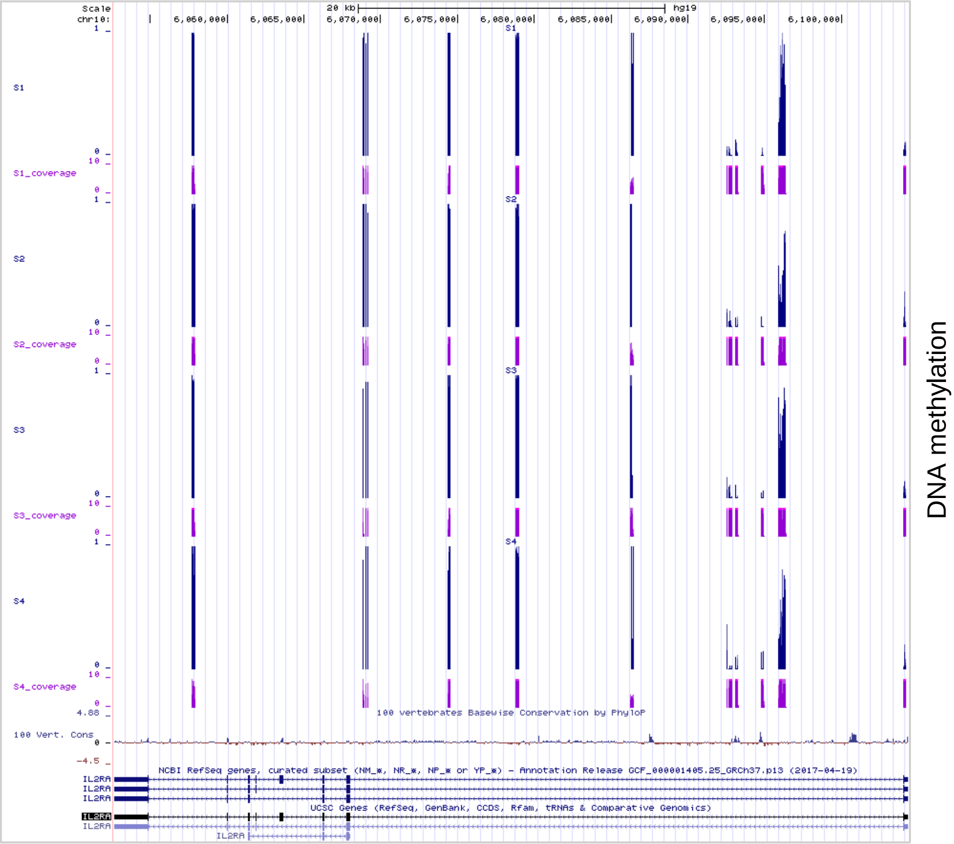

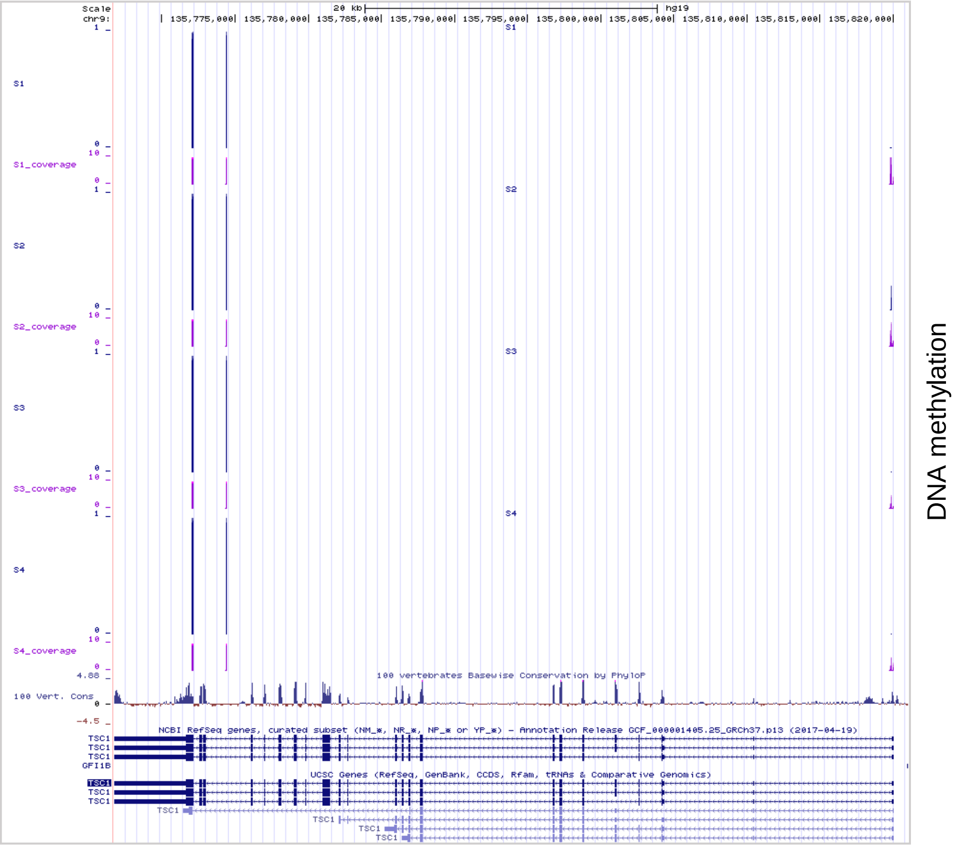

Supplemental Figure S9

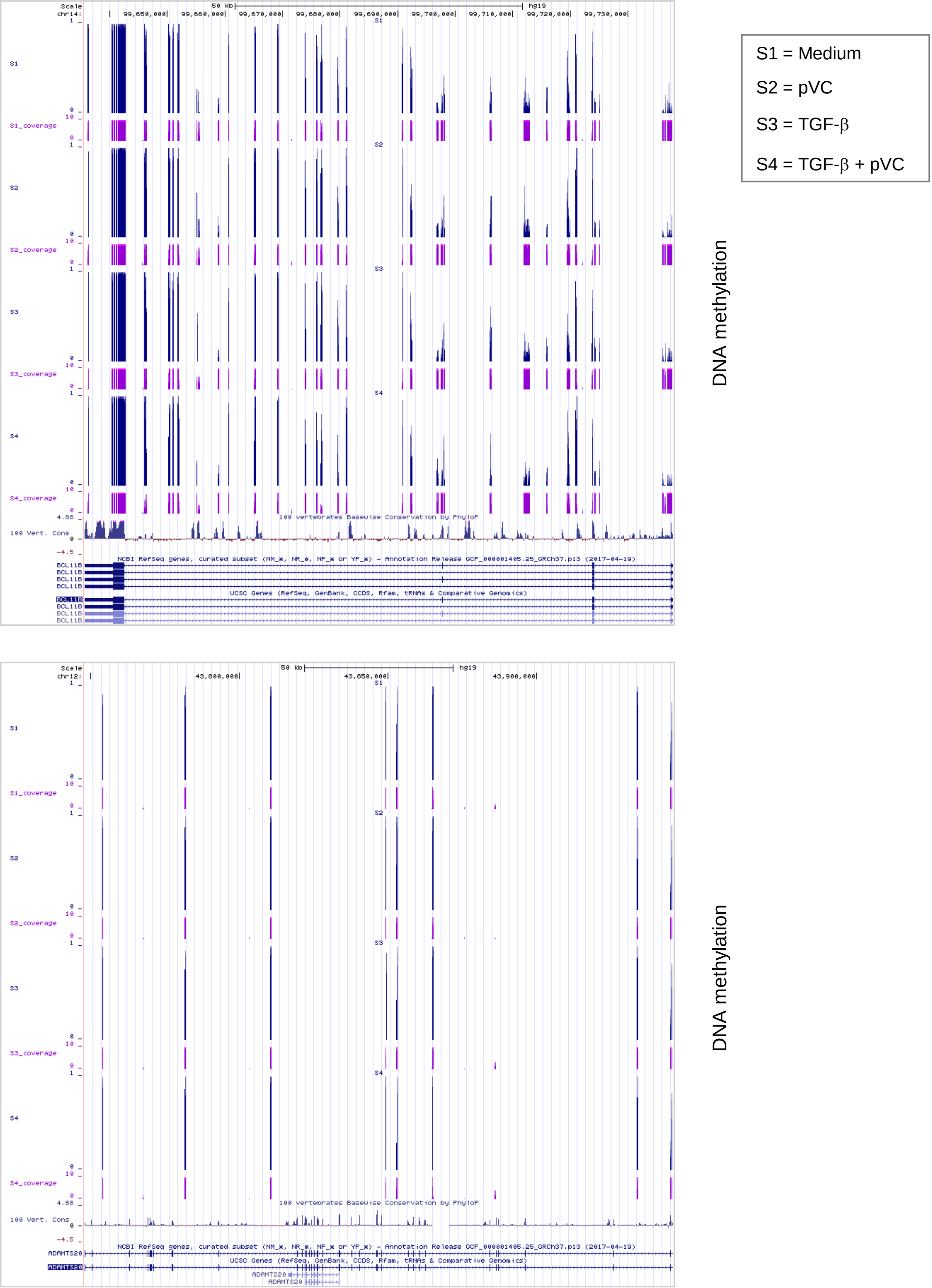

Supplemental Figure S10
